# Frequency-time coherence for all-optical sampling without optical pulse source

**DOI:** 10.1038/srep34500

**Published:** 2016-09-30

**Authors:** Stefan Preußler, Gilda Raoof Mehrpoor, Thomas Schneider

**Affiliations:** 1 Insitut für Hochfrequenztechnik, Technische Universität Braunschweig, 38106 Braunschweig, Germany

## Abstract

Sampling is the first step to convert an analogue optical signal into a digital electrical signal. The latter can be further processed and analysed by well-known electrical signal processing methods. Optical pulse sources like mode-locked lasers are commonly incorporated for all-optical sampling, but have several drawbacks. A novel approach for a simple all-optical sampling is to utilise the frequency-time coherence of each signal. The method is based on only using two coupled modulators driven with an electrical sine wave. Since no optical source is required, a simple integration in appropriate platforms, such as Silicon Photonics might be possible. The presented method grants all-optical sampling with electrically tunable bandwidth, repetition rate and time shift.

Analog-to-digital conversion (ADC) is essential for precise transformation and processing of information carried by electromagnetic signals into their binary correspondent. In computer technology, the powerful software improvement has kept pace with more complex hardware development. This is not the case in electronic systems, since the ADC and sampling rate of signals is a limiting factor for the performance. With the rapid growth of photonic technologies, there has been a great interest in reconciling the ADC requirement with optical components during the last 25 years. The electronic ADC schemes are limited by several noise sources such as quantisation noise, thermal noise and nonlinearities^1^,^2^, motivating further experimental investigations on photonic counterparts.

According to the well-known Nyquist sampling theorem, to sample and restore a signal with maximum frequency of *f*_*max*_ Hz properly, pulses with a minimum repetition rate of 2*f*_*max*_ should be utilised. This complies a sampling frequency of *f*_*s*_ = 2*f*_*max*_ = 1/*t*_*s*_, where *t*_*s*_ is the time interval between two sampling points. To achieve high resolution optical sampling, different methods have been proposed. The simplest method for sampling of an optical signal is to utilise a fast photodetector in combination with sample-and-hold circuits. However, the signal is completely sampled in the electrical domain and therefore restricted by the bandwidth of the electronic components. Another approach is based on a multiplication between the signal and a train of short laser pulses (see the Methods for details). The multiplication can be carried out in a modulator, driven by the electrical signal^1^. For this method the sampling rate is restricted by the bandwidth of the modulator. Faster sampling is achieved by multiplication in a nonlinear element^3^,^4^. A temporal magnifier such as a time lens can be used for very short optical waveforms^5^. All mentioned techniques require a stable short-period optical pulse source.

Conventionally, a mode-locked laser (MLL) is utilised as the pulse source to perform optical sampling, where the sampling rate depends on the repetition rate of the MLL. The main drawback of MLLs is that they are not flexible to be integrated into an electronic system. Additionally, the repetition rate of the pulses, and the sampling frequency depends on the optical path length of the MLLs cavity. Therefore, a tuning of the sampling frequency is rather complicated and only possible in a limited range. Furthermore, for a parallelisation of the sampling in time, tunable delay lines for each branch and an exact calibration is required. For a parallelisation in the frequency domain, interleaved pulses of different wavelengths are necessary^6^.

In this paper, we demonstrate a simple all-optical sampling, without requiring any pulse source. All sampling parameters can be tuned in the electrical domain easily and quickly and a parallelisation of sampling in time and frequency domain is straight forward.

## Theory

**Figure 1:**
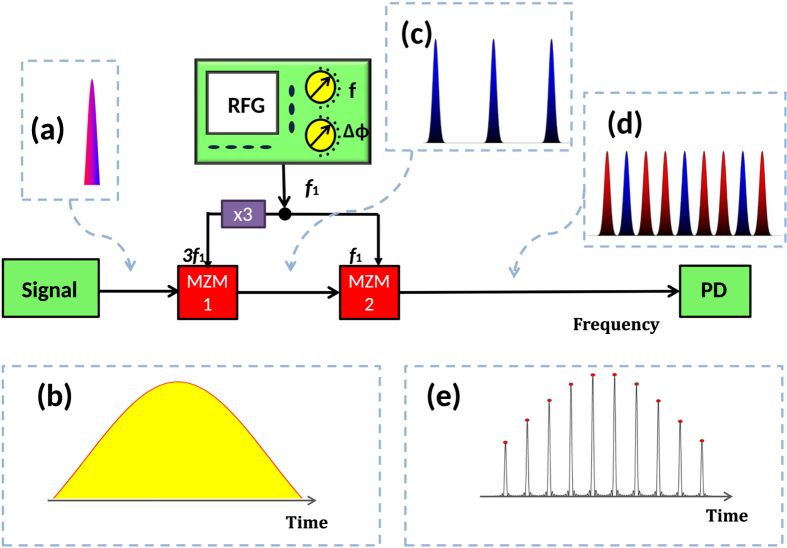
Basic principle of optical sampling based on frequency-time coherence. The insets (**a**,**b**) show the input signal in the frequency and time domain, respectively. In the first Mach-Zehnder modulator (MZM1) the spectrum of the input signal is tripled (inset (**c**)). The second modulator leads to the spectral components shown in red in inset (**d**). In order to adapt the sampling parameters to the signal, the frequency as well as the phase of the sinusoidal wave generated by the radio frequency generator (RFG) can be changed. The result is a convolution of the input spectrum with a nine-line rectangular frequency comb, corresponding to an optical sampling with a Nyquist pulse sequence in the time domain (inset (**e**)). The sampling point is the integration of the sequence in the time-interval of the repetition rate, which can be carried out with a low bandwidth photodiode (PD).

The method is based on a convolution between a frequency comb and the signal spectrum in two coupled modulators. A similar technique can be used for the demultiplexing of Nyquist channels^7^. The basic principle of all-optical sampling based on frequency-time coherence is depicted in Fig. 1. For this sampling just two coupled optical Mach-Zehnder modulators (MZM) are required. The sinusoidal input frequency *f*_1_ is generated by a radio frequency (RF) generator and can be changed in frequency and phase. This input frequency is tripled in a frequency tripler and used to drive the first modulator (MZM1). The second modulator (MZM2) is directly driven with *f*_1_. The bias voltage and RF power of both modulators are adjusted in such a way that just two new sidebands with equal power and phase as the carrier are generated^8^. The lengths of the electrical and optical connections have to be compensated, which can be done by an additional phase shifter either in the *f*_1_ or 3*f*_1_ branch (not shown). For the sake of simplicity, it is assumed that the optical input signal has a band limited spectrum with a Gaussian-like shape and a frequency and time representation as shown in Fig. 1a,b, respectively. The first modulator simply triples the spectrum as can be seen in Fig. 1c. The frequency separation between the spectra is defined by 3*f*_1_. The second modulator leads again to two new copies of the input spectrum around each carrier. Since three input carriers are present, the results are the six red copies of the input spectrum, shown in Fig. 1d. As a result, the output spectrum consists of nine equal copies of the input spectrum, with a frequency separation of *f*_1_. Since the modulators were adjusted in a way that the sidebands have the same amplitude as the carrier, the nine copies are almost equal to each other. Beside two coupled modulators, just one modulator driven with 4 equidistant input frequencies can be used. But in this case, the maximum achievable bandwidth is lower. The obtained output spectrum can be seen as a convolution between a nine-line rectangular frequency comb and the input spectrum. This convolution corresponds to a multiplication between a sinc-pulse sequence and the time signal in the time domain, as can be seen in Fig. 1e. The result is an optical sampling of the time domain signal by a sinc-pulse sequence. The same can be expressed mathematically by the equation:


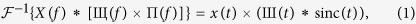


where 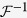 is the inverse Fourier transform and 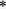 depicts the convolution. Here 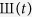 is the time domain representation of an unlimited frequency comb, 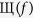,


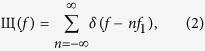


and the rectangular function Π(*f*) is defined along the bandwidth of the comb in frequency domain


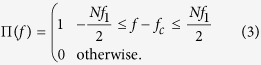


*f*_1_ denotes the frequency spacing between the comb lines, with *N* the total number of lines, determining *B* = *Nf*_1_ as the required bandwidth for the rectangle and *f*_*c*_ as the carrier frequency. This definition leads to an odd number of lines in the comb. For an even number of comb lines, the time domain representation is a sinc-pulse sequence as well^8^.

If the input signal were a Dirac delta function, i.e. *X*(*f*) = *δ*(*f*), the time response would be *x*(*t*) = 1. Therefore, if the input to the two modulators were a narrow linewidth optical wave, the output would be a rectangular nine-line frequency comb, or a sinc-pulse sequence in time domain. The repetition time of the sequence corresponds to the inverse of the frequency spacing between the lines, or the RF frequency applied to the modulator *t*_*s*_ = 1/*f*_1_. Accordingly, the inverse Fourier transform of a multiplication between a frequency comb and a rectangular function leads to a sinc-pulse sequence^8^,^9^,^10^:


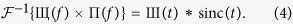


Due to reciprocity of the Fourier transform, a multiplication in frequency corresponds to a convolution in time domain, so that the right side of Equation (4) is the convolution of the two functions in time domain. Having the function definitions in time domain as


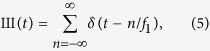



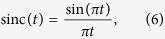


the convolution can be written as^8^:


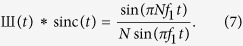


Generally, the resulting Nyquist sequence of Equation (4) is^8^


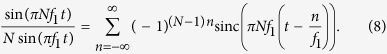


Hence, the Nyquist pulse sequence is the unlimited sum of single sinc-pulses, each of which is shifted by *n*/*f*_1_ and unlimited in time domain and correspondingly non-causal. The interference-free sum of these unlimited pulses is only possible due to the orthogonality of the pulses to each other, if the time shift corresponds to *k*/(*Nf*_1_) where *k* is an integer.

Suppose an arbitrary signal *X*(*f*) in frequency domain with *x*(*t*) being the signal in the time domain. The convolution of this signal in the frequency domain with a rectangular frequency comb corresponds to a multiplication between the signal and the Nyquist pulse sequence in time domain:


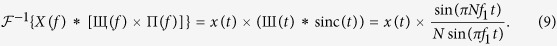


The sampled signal *x*_*N*_(*t*) is achieved by the right side of equation (9). With the order reversed, the Fourier transform of the sampled signal can be found as


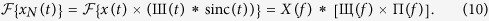


As a result, the convolution of the signal spectrum with the rectangular comb through modulation by the two coupled modulators (as shown in Fig. 1), is equal to a multiplication of an externally generated sinc-pulse sequence by the time domain signal (as in Fig. 6b).

## Experiment

**Figure 2:**
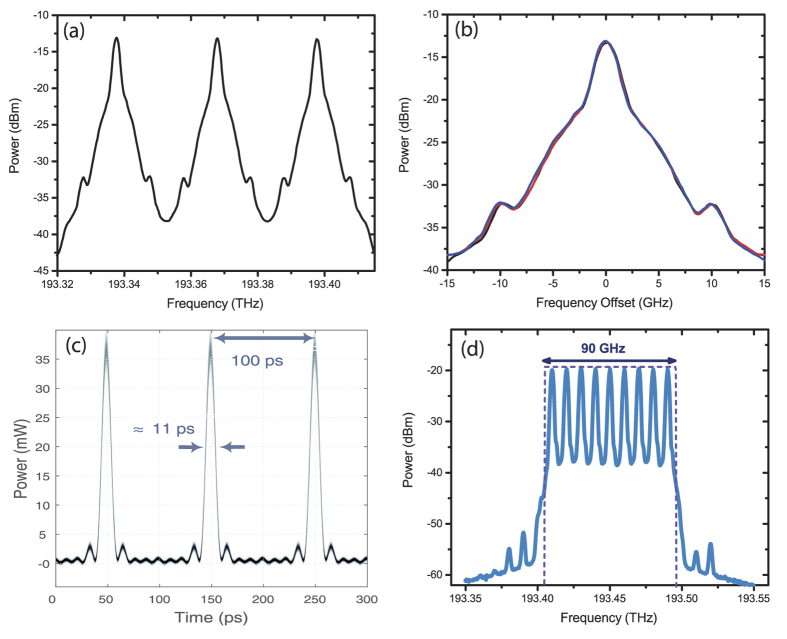
(**a**) Optical spectrum after the first modulator, driven with 30 GHz, if a 10 Gbit/s pseudo random bit sequence is used as the input signal. (**b**) illustrates the overlapping of all copies of the input spectrum; (**c**) Generated sinc-shaped pulse sequence in time domain, if a narrow linewidth optical wave is used as the input to the two modulators; (**d**) Corresponding frequency domain representation of the 9-line spectral comb with *f*_1_ = 10 GHz. The dashed rectangle is the bandwidth of one single sinc-pulse with the same duration. The broad width of the lines is a result of the limited resolution of the used optical spectrum analyser. The linewidth is a result of the laser source and in the kHz-range, around 6 orders of magnitude smaller than the comb bandwidth.

To show the high quality of the method, we measured the convolution of the frequency comb with the input spectrum of a pseudo random bit sequence with a bit rate of 10 GBit/s for the first modulator, as described in Fig. 1c. The result can be seen in Fig. 2a. As expected, the copies show very little distortions, as additionally reported in Fig. 2b. Here all three copies were overlapped to highlight the small differences. The resolution of our optical spectrum analyser was too low to show all nine copies. So, we used a CW signal as an input to generate sinc-shaped pulse sequences as shown in Fig. 2c. The corresponding frequency domain representation, i.e. the frequency comb, can be seen in Fig. 2d. The 9 comb lines have a frequency spacing of 10 GHz. For a single pulse, this corresponds to a bandwidth of *B* = *Nf*_1_ = 90 GHz (See the dashed rectangle in Fig. 2d). The time to the first zero-crossing is accordingly *τ* = 1/*B* ≈ 11 ps which approximately corresponds to the full-width at half-maximum duration of the pulses. Since both modulators are driven only with two RF frequencies generated from a single source, the generated sinc-pulse sequence is highly stable and shows a notably low noise-level. As expected, high quality sinc-shaped pulses are achieved with time jitter of less than 82 fs and low amplitude noise corresponding to a high signal-to-noise ratio level^8^. The time jitter of an MLL can be below 1 fs^11^. The time jitter and noise level were measured with a MLL based commercial optical sampling scope with a jitter of ≤100 fs, as stated in the data sheet. Thus, it might be possible that the measured jitter is that of the commercial device. Nevertheless, in our approach possible sources of jitter are the RF-source (≈20 fs from 10–40 GHz), fluctuations of the MZM and especially fluctuations of the exposed patch cables in the proof-of-concept setup. We believe that by an integration of the method, a low jitter mostly restricted by the RF-source can be achieved. The bandwidth and repetition time of the pulses can be changed by an alteration of the RF input frequency very fast and precisely. Besides, the sampling point can be shifted regarding the signal by a phase change of the electrical signal. Hence, the sampling can be adapted to the signal and it can be scanned through it electrically. Correspondingly, all-optical sampling of an arbitrary signal is realised through the introduced approach with fully tunable sampling rate. Consequently, the technique offers flexible sampling of the signals, without requiring pulse sources.

**Figure 3:**
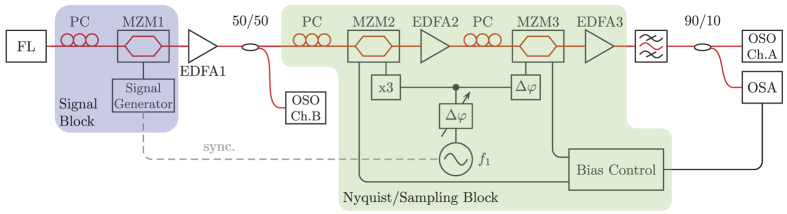
Detailed experimental setup. FL: fiber laser, PC: polarization controller, MZM: Mach-Zehnder modulator, EDFA: Erbium doped fibre amplifier, OSO: optical sampling oscilloscope, OSA: optical spectrum analyser.

Figure 3 indicates the proof-of-concept setup for the proposed all-optical sampling technique. The optical signal to sample is generated by an electrical signal in the “Signal Block”. The electrical signal is converted to the optical domain by a conventional Mach-Zehnder modulator (MZM1). A CW fibre laser (FL) operating at the wavelength of 1549.72 nm with 40 mW output power generates the optical carrier. Considering power loss caused by the MZM in the Signal Block the modulated signal is amplified by the first Erbium-doped fibre amplifier (EDFA1) afterwards. The output power of 17 dBm is split in a 3-dB coupler in order to monitor the desired optical signal at one channel of an optical sampling oscilloscope (OSO). Subsequently, the signal is fed to the “Nyquist/Sampling Block” including two coupled MZMs driven by two sinusoidal RFs generated by one generator. The output signal of MZM2 is amplified by EDFA2 to 10 dBm to compensate the power loss during modulation through MZM3. The RF *f*_1_ is produced by an RF generator. The pulsewidth as well as the point where the signal is sampled can be controlled by this modulation frequency. The first modulator in the Sampling Block (MZM2) is driven with the tripled frequency 3*f*_1_. The variable phase shifter is responsible for the time delay of the sinc-pulse train. At a frequency of 10 GHz it is capable of shifting the phase more than 380 degrees. Afterwards, the signal is split. In one branch the frequency is tripled and fed to MZM2. In the second branch the electrical path difference is adapted by a phase shifter and fed to MZM3.

For the stabilisation of the sampling, the bias voltage of the modulators is controlled in a simple feedback loop. Thus, an optical spectrum analyser (OSA) measures the maximum amplitudes of the copied spectra and a computer program adjusts the bias voltage in a way, that all these amplitudes remain equal. Output powers of the amplifiers are adjusted to constant values in order to assure the accuracy of the measurement at each step. All modulators are equipped with polarisation controllers (PC) in order to compensate the polarisation alterations along the fibres and obtain the maximum output power. The polarisation is aligned for maximum transmission. After the Sampling Block, the signal convolved with the frequency comb is amplified in EDFA3 in order to provide sufficient input power for the OSO. With the help of a 1 nm band-pass filter the out-of-band noise components are reduced.

**Figure 4:**
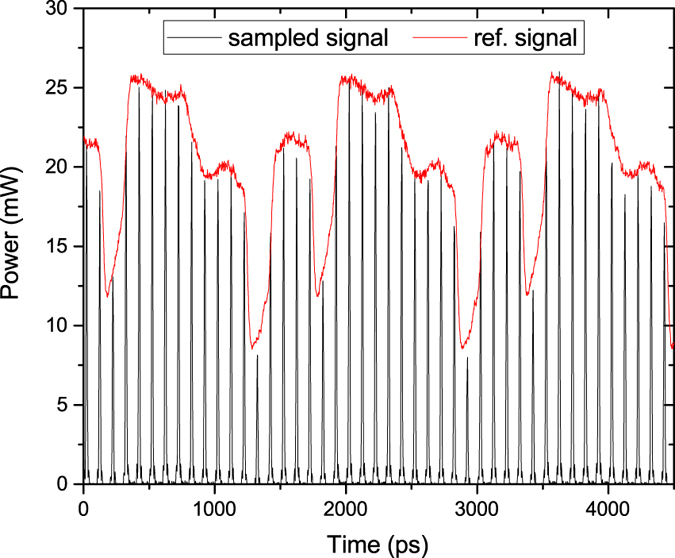
Optically sampled 16-bit data packet created by a pattern generator with a data rate of 10 Gbps. The frequency *f*_1_, applied to the coupled modulators, was 10 GHz.

We have investigated optical sampling of an arbitrary 16-bit data packet to prove the performance of the proposed technique. A pattern generator was used to create the intended data packet with a repetition rate of 100 ps. The resulting optically sampled signal can be seen in Fig. 4. A commercial OSO was used for visualisation. The red curve shows the desired signal, while the black lines correspond to the sampled signal with a rate of 100 ps. During the measurement the OSO was triggered by the sampled waveform. As can be seen, regardless of the quality of the signal from the pattern generator, it is multiplied by the pulses and sampled, respectively. Conforming to electronics, the all-optical sampling can be accomplished for periodic or non-periodic signals. Since the generated pulse-sequences possess a high quality (see Fig. 2c), we attribute the slight differences between the reference and the sampled signal to trigger problems of the OSO, and to the deficiencies of our proof-of-concept setup. The point of sampling can be varied by changing the phase of the RF frequency used to drive the modulators. Likewise, for periodical signals a simple and fast scanning through the signal is possible by an electrical phase change. For non-periodical signals higher sampling rates can be achieved by a parallelisation as will be discussed later.

**Figure 5:**
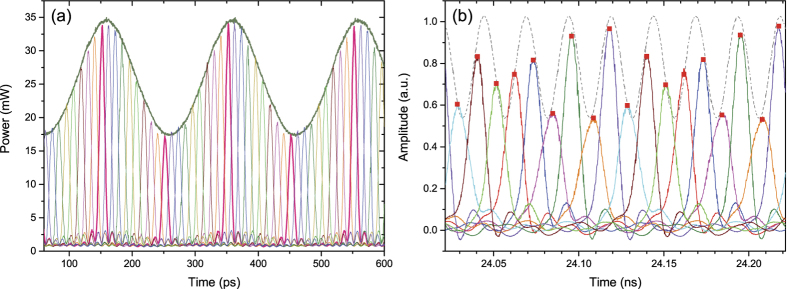
(**a**) Proof-of-concept optical sampling method. Sinusoidal waveform with a frequency of 5 GHz sampled by convolution in two coupled modulators. By an electrical phase shift of *f*_1_ and subsequent measurements, the traces with different colours were measured; (**b**) Optical sampling of a 40 GHz sinusoidal signal. The original signal which will be sampled is displayed by the gray curve. The coloured traces represent the pulse trains at different phase values and the red dots show the integrated, or the sampling values, respectively. The measurement was carried out with low bandwidth equipment, resulting in a distortion of the high-bandwidth pulse sequence. However, this does not influence the sampling. The optical sampling (multiplication) of the signal with a sinc-pulse sequence is shown in a Supplementary Movie 1.

In a proof-of-concept experiment, a 5 GHz sinusoidal waveform was used for optical sampling. To visualize the tuning of the sinc-pulse train the sampling points were time-shifted by an electrical phase shift for the frequency *f*_1_. For every trace a delay of 11 ps is added equal to the pulsewidth *τ*. Different measurements were carried out and plotted together in Fig. 5a. Moreover, the measurement of a 40 GHz sinusoidal signal can be seen in Fig. 5b, where the original waveform is represented by the dashed gray line. Therefore, the OSO was replaced by a photodiode and an oscilloscope (see Supplementary Movie 1). Within each trace of the measurement two consecutive pulses are shown and analysed. During the measurement the phase of *f*_1_ was shifted, leading to a delay of the sinc-pulse train in steps of 11.1 ps. Finally, the signal traces were recorded and integrated by software within a time window of 100 ps. The result is illustrated by the red rectangles in Fig. 5b representing the sampling points.

## Discussion

**Figure 6:**
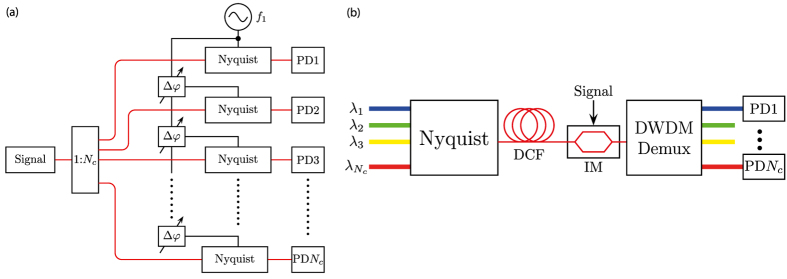
Principle configuration for parallelisation in time (**a**) and frequency domain (**b**). In (**a**) the input optical signals are fed to a 1:*N*_*c*_ coupler and transferred via optical fibres to *N*_*c*_ contiguous Nyquist sequence branches, equally delayed by a phase shift of the electrical signal. Photodiodes (PD) are utilised for the detection of the sampled optical signal. Another possibility is to carry out the sampling in the conventional way, i.e. to multiply the signal by the sinc-pulse sequence. This way one Nyquist pulse generator (two coupled modulators driven with *f*_1_ and 3*f*_1_, or one modulator driven with several coupled radio frequencies) produces the Nyquist pulse sequence and replaces the “Signal” block in (**a**). Then the sequence has to be divided into *N*_*c*_ branches and delayed in time. In each branch the sequence has to be multiplied by the signal. If the signal is in the electrical domain, this can be done with a modulator. Otherwise, a nonlinear element has to be used. Red lines are optical and black lines are electrical connections. (**b**) Principle configuration for the parallelisation in frequency domain. Different optical wavelengths 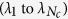 are fed to a Nyquist sequence generating block. The sequences are delayed in a dispersion compensating fibre (DCF). The multiplication in time domain is attained in an intensity modulator (IM). After demultiplexing of the sampled signals, the sampling points are detected by parallel photodiodes.

The maximum achievable sampling rate for periodical signals is restricted to four times the bandwidth of the modulator with the highest bandwidth (see the Methods section for details). For high-bandwidth non-periodic signals a parallelisation of the method is required. Then again the sampling rate can be defined by the bandwidth of the pulses *B* = *Nf*_1_ and the sampling frequency is *f*_*s*_ = *B* = 1/*τ*. This parallelisation can be achieved in time or in frequency domain. For the maximum sampling rate the next channel should be in the zero crossing of the previous one. Hence, the number of parallel channels is equal to the number of lines in the comb *N*_*c*_ = *N*. Albeit, *N*_*c*_ can be smaller than *N* if necessary. In time domain, the optical input signal is coupled through a 1:*N*_*c*_ coupler into *N*_*c*_ parallel branches as can be seen in Fig. 6a. In each branch the signal should be sampled at a fixed time delay compared to the previous one. This time delay can simply be achieved by a phase change between the electrical sine waves feeding the Nyquist sources. Thus, no optical tunable delays are required. Such tunable delay stages would only be possible by a mechanical tuning or by slow light methods^12^.

Since all sampling parameters are adjusted in the electrical domain, a fast and simple adaptation to the signal is possible. Due to parallelisation, the method grants *N*_*c*_ times larger bandwidth with slow electronic components and photodiodes for non-periodic signals.

An alternative method, is to parallelise the sampling signal in frequency domain through several wavelengths as shown in Fig. 6b. The approach is based on feeding a single Nyquist generator module with different input optical frequencies, causing generation of several frequency combs with central frequencies equal to the input optical frequencies. The following dispersion compensating fibre (DCF) delays the sinc-pulse sequences by 2*πL* × *k*_2_ × Δ*f*, where *L* and *k*_2_ are the length and group velocity dispersion of the DCF, respectively. Δ*f* is the frequency difference between the centre frequencies of the frequency combs. The delayed sequences are multiplied by the signal in an optical modulator. For higher sampling speeds, a nonlinear multiplication can be used. The sampling signals at different wavelengths are demultiplexed in an appropriate device and detected. The frequency difference could correspond to a multiple of the ITU-T frequency grid for dense wavelength division multiplexing (*n* × 50 GHz)^13^. Thus, CW lasers from optical telecommunications can be used as sources and the demultiplexing can be performed with an arrayed waveguide grating for instance.

## Conclusion

In conclusion, a method for tunable all-optical sampling without requiring a pulse source based on time-frequency coherence was presented. Two coupled modulators were used to convolve the spectrum of the optical input signal with a rectangular frequency comb, corresponding to a multiplication between the time domain representation of the signal and a sinc-pulse sequence. The parameters of the sampling points (sampling rate, bandwidth, position) are just defined by the sinusoidal input used to drive the modulators. Thus, they can be adapted to the signal fast and easily and a time shift is possible by a phase change. The sampling rate considering the maximum bandwidth of the pulses, or their minimum duration is restricted by the bandwidth of the modulators. With two intensity modulators, the maximum achievable bandwidth is four times the bandwidth of the modulator with the highest one (see the Methods for details). Therefore, a sampling rate of 400 Gs/s is possible with integrated 100 GHz modulators^14^. Sampling rates in the Tb/s range can be achieved by modulators driven with phase-locked optical frequencies^10^ and the full field can be sampled by a balanced detector^15^. An integration of the method on a silicon photonics platform, for instance, could enable small-footprint, low-cost optical sampling devices.

## Methods

### Sampling

**Figure 7:**
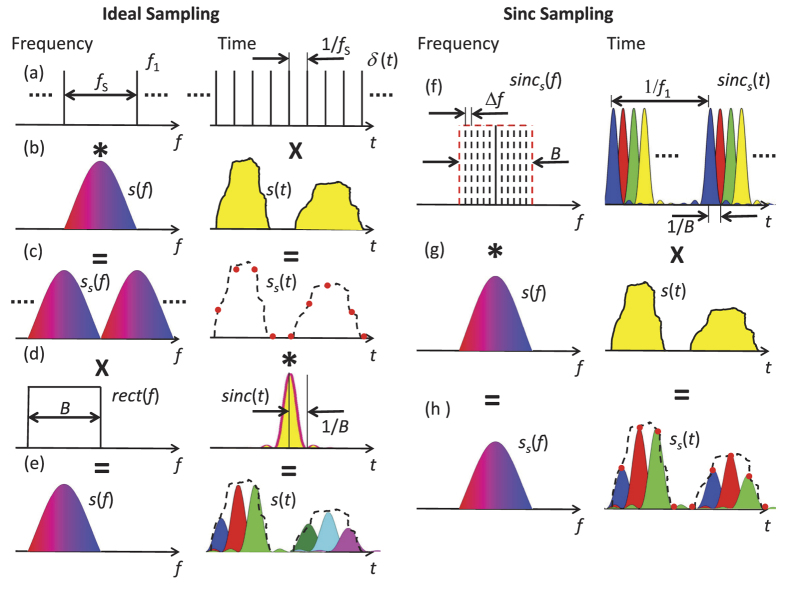
Sampling of a signal in the frequency and time domain with a Dirac-Delta function (left), corresponding to “ideal” sampling and a sinc-pulse sequence (right). The × and 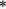 denote the signs for a multiplication and convolution, respectively. The = marks the corresponding result of the operation and *sinc*_*s*_(*t*) is the sinc-pulse sequence defined in Equation (8).

To convert a continuous signal to a digital one in time domain, a sampling of the time domain waveform is required. The analogue signal to sample *s*(*t*) (Fig. 7b) is supposed to be a band limited function with a baseband bandwidth of *B*_*s*_ and the signal can be recovered, if the sampling rate (number of samples per second) is at least twice the maximum frequency present in the signal spectrum *f*_*s*_ = 1/*t*_*s*_ > 2*B*_*s*_. Here *t*_*s*_ is the time duration between two samples, or the inverse of the sampling rate (Fig. 7a, right). The mathematical description of sampling can be found in almost every textbook of signal theory, see for instance^16^. The sampled discrete signal in time domain is (Fig. 7c, right):


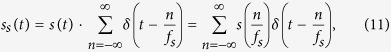


where *δ*(*t*) is an ideal Dirac delta function. In the frequency domain this corresponds to a convolution 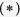 between the frequency domain representations of the signal *S*(*f*) and the Dirac delta function (Fig. 7a, left):


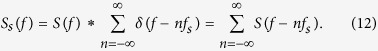


The result is an unlimited number of copies of the signal spectrum with a frequency distance of *f*_*s*_. This sampled signal can be reconstructed by a multiplication by a rectangular function in the frequency domain (Fig. 7d, left). This multiplication only filters one of the unlimited number of spectra.


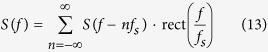


The result is the analogue signal (Fig. 7e, right):


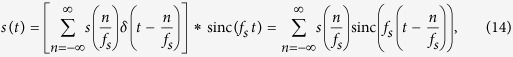


with sinc as the sinus cardinals or sinc-function, as defined above. Thus, a band limited analogue signal can be seen as a superposition of an unlimited number of sinc-pulses weighted with the sampling value and time shifted by the sampling time.

The sampling with the method presented here leads to (Fig. 7h, left):


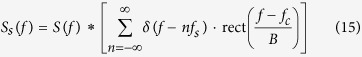


in the frequency domain, with *f*_*c*_ as the carrier frequency of the optical wave. So, the copies of the spectrum are not unlimited as for the sampling with a Dirac Delta function, but restricted by the number of lines in the comb *N*. In the frequency domain each copy of the spectrum can at the most be as broad as the frequency spacing between the comb lines. For this reason, this restricts the sampling rate to *f*_*s*_ = *f*_1_ for the sampling of a non-periodical signal. The time spacing between the sampling points is equal to the repetition rate of the sinc-pulse sequence. Whereas for a periodical signal, the sinc-pulse sequence can be shifted in time domain to any point. For parallelised sampling, in each branch the sampling points are taken with the repetition rate of the sequence. But due to prallelisation and time shift, the minimum time between two sampling points can be the time between the maximum of the sinc-pulse and the first zero crossing at *τ* = 1/*B*. Thus, for periodical signals and for a parallelised sampling it can be written that *B* = *f*_*s*_. In time domain it follows (Fig. 7h, right):


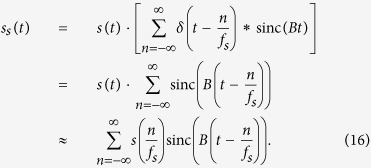


By the comparison with Equation (14) it can be seen that the method grants access to the sampling values *s*(*n*/*f*_*s*_) by an integration of the sinc-pulse sequence over the time interval 1/*f*_1_. This integration can be carried out with a photodiode with a restricted bandwidth of *f*_1_ = *B*/*N*.

### Tunability

According to the left side of Equation (4), the frequency representation of the sinc-pulse sequence is a rectangular frequency comb. The individual comb lines have to have the same or a linear dependent phase. A simple method to generate such a frequency comb is to use two coupled intensity modulators. Both modulators are adjusted in such a way that the first order sidebands have the same amplitude as the carrier and higher order sidebands are suppressed. If the DC bias and the RF signal voltages applied to a single modulator are *V*_*B*_ and *v*_*s*_ cos(*ω*_*s*_*t*), respectively, the expression for the output field can be written as^17^


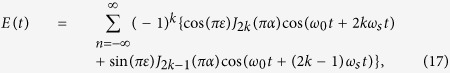


where *J*_*k*_ is the Bessel function of the first kind and order *k*, *ε* = *V*_*B*_/*V*_*π*_ and *α* = *v*_*s*_/*V*_*π*_, in which *V*_*π*_ is the half-wave voltage of the modulator. Contrary to phase modulators, the amplitude of the carrier, first-order sidebands and higher-order sidebands in intensity modulators can be adjusted by two parameters, i.e. the RF driving voltage *α* and the DC bias *ε*. If Equation (17) is reduced to the spectral components of interest, i.e. the carrier and the first order sidebands, the electric field at the output of the modulator can be written as^8^:


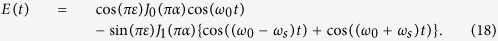


**Figure 8:**
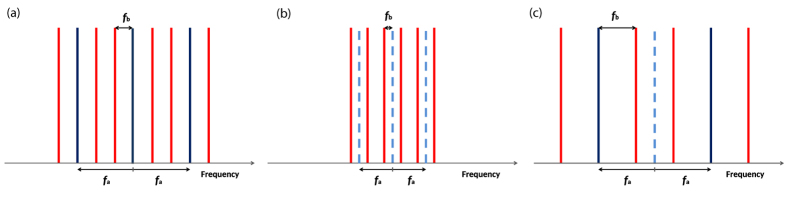
(**a**) 9-line frequency comb, the 3 blue spectral lines are generated by the first modulator, whereas the second modulator generates the red lines; (**b**) Both modulators operate in the suppressed carrier regime (dashed lines). The result is a 6 line frequency comb; (**c**) The first modulator (blue lines) operates in the suppressed carrier regime (dashed blue line) and the second modulator generates the red lines.

If two intensity modulators are used and both have just one input frequency, then there are three possibilities of generating a rectangular frequency comb as illustrated in Fig. 8. If the carrier is not suppressed as in Fig. 8a, the modulators generate *N* = 9 lines. The first modulator is driven with the frequency *f*_*a*_. Then, the modulator generates the three blue lines. In the second modulator these lines are re-modulated with the frequency *f*_*b*_ = 1/3*f*_*a*_ to produce the red lines. If at least one of the modulators can be driven with a maximum frequency of *f*_*a*_ = 40 GHz, the maximum bandwidth is *B* = *N* × *f*_*b*_ = 120 GHz, which corresponds to a pulse width (FWHM) of around 8 ps. If both modulators are driven in the suppressed carrier regime (Fig. 8b), an even number of lines is produced (*N* = 6) but with a maximum bandwidth of *B* = 60 GHz (17 ps) since *f*_*b*_ = 1/4*f*_*a*_. In Fig. 8c just the carrier of the first modulator is suppressed. Therefore, an even number of lines is produced. Here the second modulator has to be driven with *f*_*b*_ = 2/3*f*_*a*_ and the maximum bandwidth is *B* = 160 GHz, corresponding to 6 ps pulses. Of course, the first modulator can be driven with the lower and the second one with the higher frequency, causing the same results. In consequence, the maximum possible bandwidth with two coupled MZMs corresponds to 4 times the bandwidth of the modulator with the highest bandwidth.

The repetition period of the Nyquist pulses, and therefore the number of parallel branches or the wavelength for sampling, is defined by the number of lines. Nine lines can be produced if just one modulator is driven with a four-frequency input signal. In effect, each odd number *N* of lines can be produced with just one modulator if the RF-signal driving the modulator consists of (*N* − 1)/2 equally spaced frequency lines, for an even number of lines the number of RF is *N*/2. These equally spaced frequency lines in the RF domain can be produced by a mixing of higher harmonics, or by an arbitrary waveform generator for instance, even though the maximum bandwidth of this method is restricted by around twice the bandwidth of the used modulator. Another possibility to increase the number of lines is the usage of more than two modulators. However, the maximum bandwidth which can be achieved does not increase much with the number of additional modulators. Higher bandwidths can be achieved if higher order sidebands are used. Since the carrier and the first order sidebands are out of phase, this requires an active suppression of these components. One possibility to achieve Nyquist pulses with very broad bandwidths is the filtering of the frequency comb of a mode-locked source^12^. But, due to the non-ideal bandwidth of optical filters, the generated pulses have no sinc shape and the pulse parameters cannot be adapted to the sampled signal easily. Instead of filtering a whole comb as rectangular as possible, single comb lines can be filtered. These lines are the initial lines for a subsequent modulation. The initial lines are responsible for the arbitrary bandwidth, not limited by modulators, and the subsequent modulation enables an arbitrary repetition period of the pulses. As a mode-locked source, a fibre fs-laser, or at much lower costs and being simpler in usage, a comb generator can be used. In proof-of-concept experiments, sinc-shaped Nyquist pulses with a bandwidth of around 300 GHz have been generated by this method^10^. However, bandwidths in the THz-range and correspondingly femtosecond pulses should be possible.

## Additional Information

**How to cite this article**: Preußler, S. *et al*. Frequency-time coherence for all-optical sampling without optical pulse source. *Sci. Rep.* **6**, 34500; doi: 10.1038/srep34500 (2016).

## Supplementary Material

Supplementary Movie S1

Supplementary Information
