# Supplementary material for: Frequency-time coherence for all-optical sampling without optical pulse source
Source: Sci Rep. 2016 Sep 30;6:34500. doi: 10.1038/srep34500 (PMC5043285; doi:10.1038/srep34500)
Supplement: Supplementary Information [file srep34500-s2.pdf]

Frequency-time coherence for all-optical sampling without optical pulse source

Stefan Preussler[1], Gilda Raoof Mehrpoor[1], and Thomas Schneider[*]

[1]*Insitut für Hochfrequenztechnik, Technische Universität Braunschweig,*

38106, Braunschweig, Germany
[*]*Corresponding author: thomas.schneider@ihf.tu-bs.de*

Supplementary Movie 1

This video shows the optical sampling of a 40 GHz sinusoidal signal (shown in red) with a sinc-pulse sequence and monitored with a low bandwidth photodiode (50 GHz, see Fig.5b in the paper). The yellow line represents the integrated values of the sinc pulse sequence over the repetition rate of the pulses. The scanning through the signal is accomplished by changing the phase of f_1 per hand. We address the differences to a phase alteration of the proof-of-concept setup during the measurement.
